# Brain-Derived Neurotrophic Factor (BDNF) in Mechanisms of Autistic-like Behavior in BTBR Mice: Crosstalk with the Dopaminergic Brain System

**DOI:** 10.3390/biomedicines11051482

**Published:** 2023-05-19

**Authors:** Tatiana Ilchibaeva, Anton Tsybko, Marina Lipnitskaya, Dmitry Eremin, Kseniya Milutinovich, Vladimir Naumenko, Nina Popova

**Affiliations:** Federal Research Center Institute of Cytology and Genetics, Siberian Branch of Russian Academy of Sciences, Prospekt Akad. Lavrentyeva 10, 630090 Novosibirsk, Russia; antontsybko@bionet.nsc.ru (A.T.); m.lipnitskaya@g.nsu.ru (M.L.); eremin@bionet.nsc.ru (D.E.); milutinovic.ksenija@yandex.ru (K.M.); naumenko2002@bionet.nsc.ru (V.N.); npopova@bionet.nsc.ru (N.P.)

**Keywords:** BDNF, TrkB, dopamine system, autistic-like behavior, BTBR mice, dopamine receptor, AAV-induced overexpression

## Abstract

Disturbances in neuroplasticity undoubtedly play an important role in the development of autism spectrum disorders (ASDs). Brain neurotransmitters and brain-derived neurotrophic factor (BDNF) are known as crucial players in cerebral and behavioral plasticity. Such an important neurotransmitter as dopamine (DA) is involved in the behavioral inflexibility of ASD. Additionally, much evidence from human and animal studies implicates BDNF in ASD pathogenesis. Nonetheless, crosstalk between BDNF and the DA system has not been studied in the context of an autistic-like phenotype. For this reason, the aim of our study was to compare the effects of either the acute intracerebroventricular administration of a recombinant BDNF protein or hippocampal adeno-associated-virus–mediated BDNF overexpression on autistic-like behavior and expression of key DA-related and BDNF-related genes in BTBR mice (a widely recognized model of autism). The BDNF administration failed to affect autistic-like behavior but downregulated *Comt* mRNA in the frontal cortex and hippocampus; however, COMT protein downregulation in the hippocampus and upregulation in the striatum were insignificant. BDNF administration also reduced the receptor TrkB level in the frontal cortex and midbrain and the BDNF/proBDNF ratio in the striatum. In contrast, hippocampal BDNF overexpression significantly diminished stereotypical behavior and anxiety; these alterations were accompanied only by higher hippocampal DA receptor D1 mRNA levels. The results indicate an important role of BDNF in mechanisms underlying anxiety and repetitive behavior in ASDs and implicates BDNF–DA crosstalk in the autistic-like phenotype of BTBR mice.

## 1. Introduction

Although autism spectrum disorders (ASDs) are the most commonly diagnosed neurodevelopmental disorders [1], their etiology remains largely unclear. ASD patients share a similar behavioral profile (dysfunction of communication and of social interaction as well as repetitive behavior), but the pathobiological processes resulting in ASD show considerable heterogeneity. On the other hand, impairments of neuroplasticity are widely accepted as the main cause of ASD. These changes in neuroplasticity processes may arise from disturbances in synaptic function [2,3] and in activities of neurotransmitter systems [4,5,6].

Despite the obvious importance of dopamine (DA) in the modulation of learning, reward, emotional control, and executive functions, the participation of the DA system in the mechanism of ASD has not been investigated substantially. ASD patients demonstrate diminished responses to rewarding stimuli, especially those associated with social reinforcement [7,8]. At the same time, the degree of social dysfunction correlates in young and adult patients with reward-related learning disabilities [9,10]. fMRI data show that the ventral striatum in autistic patients is not activated in response to social and monetary reward [11,12,13,14]. These studies highlight decreased sensitivity of the mesolimbic reward neural network and suggest that an imbalance of DA neurotransmission may underlie the reward stimuli’s diminished relevance observed in autism. Furthermore, in recent years, an association of autism with a number of deletions and single-nucleotide polymorphisms in DA receptors [15,16,17], DA transporter (DAT) [18,19,20], and enzymes of DA metabolism [21,22] was revealed. Moreover, DA neurons derived from reprogrammed-induced pluripotent stem cells from autistic patients have defects in calcium signaling and neurite outgrowth [23]. These pieces of evidence have given rise to the DA hypothesis of autism, which postulates that disturbances in the nigrostriatal pathway and mesolimbic pathway lead to autism-like symptoms [24,25].

Neurotrophic factors play one of the central roles in neuroplasticity processes and are inevitably involved in the regulation of both normal and pathological behavior [26,27,28]. Being widespread in the central nervous system, brain-derived neurotrophic factor (BDNF) is a key transducer of neuroplasticity processes because it is critically involved in the control of neuro- and synaptogenesis, protection and maintenance of various populations of neurons, and the induction of long-term potentiation and is the most important regulator of memory and thinking [29,30,31]. The relation of BDNF with autism has been evaluated in a number of studies on ASD patients. The majority of such articles are devoted to the assessment of the peripheral BDNF level in serum or plasma. Meta-analyses have uncovered a positive association between autism and high blood BDNF levels [32,33,34,35,36]. Nevertheless, some authors report reduced serum BDNF levels in autistic patients [37,38,39,40,41,42]. It should be noted that there are no data on any correlation between BDNF concentrations in the central nervous system and human blood. Only one study has been published about BDNF and proBDNF mRNA and protein levels in postmortem brain samples from autistic patients [43]. In that study by Garcia and coauthors [43], an increase in the total BDNF level was demonstrated in the fusiform gyrus of ASD patients. At the same time, the proBDNF level was also found to be elevated, implying problems in BDNF maturation [43]. Without more detailed research on BDNF expression in the brain, it is difficult to make conclusions about the correlation between autism and peripheral BDNF levels. In fact, peripheral BDNF levels can depend on diet and physical activity [44], circadian rhythms [45,46], and gut microbiota [47], thereby complicating the interpretation of the findings.

Crosstalk between BDNF and the DA system is well known. It has been shown that BDNF is a key neurotrophic factor for DA neurons in the substantia nigra (SN) [48] and not only determines the neuronal phenotype but also stimulates DA secretion [49,50]. Heterozygous BDNF+/− knockout mice exhibit accelerated aging of DA neurons in the SN and striatum [51]. A similar effect is achieved by the downregulation of BDNF with antisense oligonucleotides [52]. BDNF–receptor TrkB signaling is activated in response to stress or damage to DA neurons [53,54,55] and seems to be necessary for their protection [56]. For instance, DA neurons expressing TrkB are more resistant to a toxin called 1-methyl-4-phenyl-1,2,3,6-tetrahydropyridine (MPTP) [57]. In experimental models of Parkinson’s disease, a therapeutic effect has been achieved with BDNF overexpression [58,59,60] or with BDNF administration [61,62,63]. Additionally, BDNF–TrkB signaling is important for the normal functioning of the mesolimbic DA pathway, its adaptation to stress, and the formation of a drug response [64,65]. On the other hand, the crosstalk between BDNF and the DA system in autism has not been studied yet. At present, there is only one paper pointing to such a link. Heterozygous D2^+/−^ knockout mice exposed to early postnatal stress develop symptoms similar to those in autism; this problem is accompanied by BDNF and TrkB underexpression in the dorsal striatum [66]. In this context, administration of TrkB agonist 7,8-dihydroxyflavone (7,8-DHF) reverses the behavioral abnormalities [66].

Because the majority of ASD cases have an idiopathic nature, the most attractive and widely used ASD animal model is the inbred BTBR T+Itpr3tf/J (BTBR) strain of mice [67,68]. BTBR mice consistently demonstrate significant manifestations of autistic-like behavior, which can be divided into two main domains: (1) disturbances of social interaction and communication and (2) high stereotypy [67,69]. In addition to behavioral impairments, BTBR mice share neuroanatomical features with ASD patients [70]; in addition, appreciable similarities can be found in the disturbances of many signaling pathways [68]. It is reported that BTBR mice have considerable aberrations in the brain DA system. In particular, their DA receptor D2 activity is significantly reduced and the mesolimbic pathway is barely activated [71]. In addition, a significant decrease in the expression of an enzyme taking part in DA synthesis, tyrosine hydroxylase (TH), has been revealed in the SN and ventral tegmental area, and the same is true for DAT in the dorsal striatum [72]. Intranasal administration of DA not only raises the TH level in the striatum of BTBR mice but also significantly increases their social interest and object recognition [72]. Reduced BDNF mRNA expression has been found in the hippocampus [73,74] and the frontal cortex [74] of BTBR mice. Diminished BDNF and TrkB protein levels have also been observed in the hippocampus [75]. It is noteworthy that the treatment of hippocampal slices with BDNF provokes excitatory postsynaptic potentials in control C57BL/6J mice but not in BTBR mice [75]. In old BTBR mice (15 months of age), the BDNF protein amount is decreased both in the hippocampus and in the frontal cortex [76], whereas on embryonic day 18, the BDNF level is significantly elevated [77].

Several studies suggest that an enriched environment [78], neuromodulator fingolimod [79], palmitoylethanolamide [80], and β-carotene [81] raise BDNF or TrkB mRNA levels in the hippocampus of BTBR mice. In a study by Segal-Gavish and coauthors [82], human mesenchymal stem cells were transplanted into a lateral ventricle of BTBR mice; this procedure lowered stereotypy and improved social behavior of the animals. These alterations were accompanied by an increase in BDNF concentrations in the hippocampus and frontal cortex [82]. Similarly, when amniotic epithelial cells are transplanted into a brain ventricle of BTBR mice, an improvement in behavior is accompanied by BDNF and TrkB overexpression in the hippocampus [83]. Nevertheless, in addition to BDNF, amniotic epithelial cells secrete VEGF, SDF-1, IL-6, GDNF, and NGF [84,85], and the latter is secreted four times more than BDNF [84]. Human mesenchymal stem cells also secrete neurotrophins NGF, NT-3, and NT-4 [86]. Therefore, transplantation of cells performing paracrine secretion of various neurotrophic and growth factors is a rather nonspecific approach that does not provide knowledge about a possible therapeutic action of BDNF on behavioral disturbances in BTBR mice.

It is known that ASD is characterized by an increase in brain volume due an abnormal cortical overgrowth pattern and by increases in size, spine density, and neuron population that leads to the dysregulation of postnatal synaptic pruning and results in a huge variety of forms and degrees of signal-over-noise discrimination losses [87]. On the other hand, BTBR mice also demonstrate a similar spectrum of neuromorphological abnormalities [88,89,90,91,92,93] including the absence of corpus callosum [94], known for ASD patients [95]. Thus, we can hypothesize that a BDNF-induced increase in neurogenesis and axonal growth may exert diverse effects on autistic-like behavior.

The aim of our study was to compare the effects of the intracerebroventricular (i.c.v.) administration of the BDNF protein and intrahippocampal adeno-associated virus (AAV)-mediated BDNF overexpression on the autistic-like behavior (locomotion, exploration, and anxiety in open field and elevated plus maze tests, stereotypical behavior in the marble burying test, social behavior in the three-chambered social test, and cognition in the novel object recognition test) and expression of key DA (D1 and D2 receptors, catechol-O-methyltransferase, tyrosine hydroxylase, and dopamine transporter) and BDNF (TrkB and p75 receptors, BDNF and proBDNF) genes elements in the brains of BTBR mice.

## 2. Materials and Methods

### 2.1. Animals

**Figure 1:**
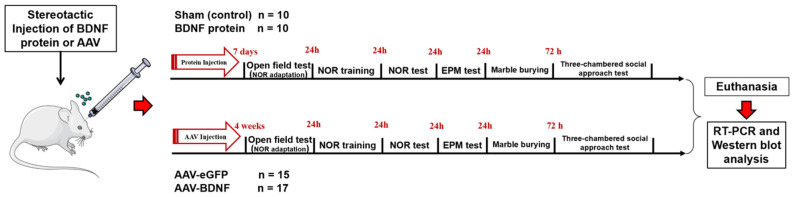
The experimental design. NOR: novel object recognition, EPM: elevated plus maze.

Experiments were performed on specific pathogen-free adult (P60, 25 ± 1 g) male mice of the BTBR inbred strain. The mice were housed at the Center for Genetic Resources of Laboratory Animals at the Institute of Cytology and Genetics, the Siberian Branch of the Russian Academy of Sciences (ICG SB RAS) (unique identifier RFMEFI62119X0023) under standard laboratory conditions on a 12/12 h light/dark cycle with water and food available ad libitum. The source of the mice was Charles River Laboratories. Two days before the behavioral experiment, the mice were isolated by placement into individual cages to remove group effects. In the first experiment (BDNF i.c.v. injection), the number of animals in each group was 10; in the second experiment (AAV-mediated *Bdnf* overexpression), the number of mice was 15 in the control group and 17 in the experimental group (Figure 1). All surgical procedures were performed under anesthesia, and every effort was made to minimize the suffering of the animals.

### 2.2. BDNF i.c.v Injection

Recombinant BDNF protein (Sigma-Aldrich, St. Louis, MS, USA) was diluted in sterile water and injected in a dose of 300 ng into the left lateral ventricle of each mouse (AP: −0.5 mm, ML: −1.0 mm, DV: 2 mm; http://labs.gaidi.ca/mouse-brain-atlas/?ml=-1&ap=-0.5&dv=2, accessed on 1 September 2021). Before this procedure, the animals were anesthetized for 20–30 s with isoflurane. Sterile water was injected as a control (a sham group). The volume of i.c.v. administered solutions was 3 μL. Behavioral testing was started 7 days after the BDNF injection.

### 2.3. A Cell Line

HEK 293FT cells (ATCC cat. # PTA-5077) were used to produce recombinant AAV (rAAV) vectors. The cell line was routinely maintained in DMEM containing 10% (*v*/*v*) of FBS (F2442, Sigma-Aldrich, St. Louis, MS, USA) and 100 U/mL penicillin/streptomycin (P4333, Sigma-Aldrich, Darmstadt, Germany) at 37 °C in a humidified atmosphere of 95% air and 5% CO_2_. The cells were split at 70% confluency, and the culture medium was refreshed every 2 or 3 days.

### 2.4. Production of rAAV Vectors

The cDNA encoding mouse *Htr1a* was cloned into the pAAV-Syn-eGFP vector. The packaging of DNA of the pAAV-Syn-Bdnf-eGFP plasmid or of the control pAAV-Syn-eGFP plasmid into rAAV capsids was performed using co-transfection with plasmids AAV-DJ and pHelper (Cell Biolabs, Inc., San Diego, CA, USA). Viral particles were harvested in 48 h according to the protocol described by Grimm and coauthors [96]. The amount of the obtained viral particles was determined with real-time quantitative PCR with primers F 5′-cctggttgctgtctctttatgagg and R 5′-tgacaggtggtggcaatgc. A series of dilutions of an original plasmid of known concentration was used as standards for determining the number of viral particles. All AAV vectors employed in this study had identical genomic titers (10^9^ viral genomes per microliter).

### 2.5. Stereotaxic Microinjections

Mice were anesthetized with solution (1 mL/kg) of 2,2,2-tribromoethanol (T48402-25G, Sigma-Aldrich, Darmstadt, Germany) in 2-methyl-2-butanol (240486, Sigma-Aldrich, Darmstadt, Germany), administered intraperitoneally (i.p.). The rAAV vectors were bilaterally injected into the hippocampus at the following coordinates: AP: −1.5, ML: ± 1, DV: 2.0 and AP: −2.5, ML: ± 2.0, DV: 2.0 (according to preliminary experiments). Namely, 0.5 μL of a viral vector (10^9^ viral particles/μL) was microinjected into the site at the rate of 0.1 μL/min using a Hamilton Syringe. The syringe was left in place for 3 min and then raised slowly. After the bilateral injection of the rAAV, the incision was closed with interrupted silk sutures, and the animal was placed in a warm cage and monitored closely. Behavioral testing was performed after a 4-week recovery (Figure 1).

### 2.6. Behavioral Testing

For the assessment of locomotor activity, the open field test was carried out. A circular arena (60 cm in diameter with a plastic wall 25 cm high) was used, which was illuminated through a mat and semitransparent floor with 2 halogen lamps of 12 W each placed 40 cm under the floor [97]. A mouse was placed near the wall, and its movements were tracked for 5 min with a digital camera (Sony, Tokyo, Japan). The arena was carefully cleaned after each test. The original EthoStudio software was used for the video stream analysis frame by frame [98]. The total distance traveled, explored area of the arena, and time in the center were measured automatically. The number of rearings as a measure of exploratory behavior was assessed manually.

The evaluation of stereotypical behavior was performed with a marble burying test [99,100]. Mice were placed individually in polypropylene cages (Optimice) containing 18 clean glass marbles 1.5 cm in diameter, evenly spaced on 5 cm deep sawdust without feed or water. The ceiling was a polypropylene grid. The numbers of marbles buried by less than a half deep, by a half, and by more than a half deep were determined 30 min later.

The three-chambered social approach test was performed as described previously [101,102]. Each mouse was placed separately in a rectangular socialization device (60 × 40 × 22 cm) made of opaque plastic. The test consisted of two phases: habituation and socialization. Each test mouse was placed in the central chamber during the habituation phase to freely explore the chambers for 10 min. During the socialization phase, a new object (O) and a new mouse (S) were placed on each side of the chamber, and each experimental mouse was allowed to explore all 3 chambers for 10 min. The time spent in each chamber and the sniffing time (nose toward the cage at a distance of less than 2 cm) during each 10 min phase were recorded using the EthoStudio software [97]. The equipment was carefully cleaned after each experiment. A social preference index was calculated as the difference in time between the new mouse and the new object divided by the total time spent in the two side chambers or sniffing targets (S − O/total time).

Anxiety-related behavior was assessed using the elevated plus maze test. The setup for this test consisted of 4 arms 30 cm long and 6 cm wide connected at an angle of 90° and located at a height of 60 cm above the floor. One pair of arms had walls twenty cm high (closed arms, safe), and the other pair had no walls (open arms, potentially dangerous). The arena was illuminated from above with diffused light, which made it possible to automatically trace a dark-colored animal in the open arms [98]. Each mouse was placed at the intersection of the 2 arms, and movement was automatically recorded for 5 min. With the help of the EthoStudio software, the following parameters were automatically estimated: distance traveled (m), time (%), and explored area (%) in open and closed arms. Risk assessment behavior was evaluated as the number of head-dips and was measured manually. The apparatus was cleaned with wet and dry napkins after each test.

The novel object recognition test was used for assessment of learning and memory of mice. On a training day, two identical objects were placed in the arena at an equal distance from each other and from the walls. To eliminate the preference by the animals for certain characteristics of the objects, half of the mice of each group were presented with object X, and the other half with object Y. The animals were placed one at a time in the arena for 5 min, where they were free to interact with the objects. The mice were then returned to their Individual cages, and the arena and objects after each animal were carefully cleaned. On the test day, one of the old objects was placed in the arena along with a new one, unfamiliar to the animal. After that, the mouse was placed in the center of the arena and recorded for 5 min, during which the animal was free to explore both the new and the old object. The time of interaction with new and old objects was recorded manually in EthoStudio. The following interactions with the object were registered: sniffing (distance from the nose less than 1 cm) and standing up while leaning on the object. At the end of the test, the mouse was returned to its individual cage, and the arena and objects were thoroughly cleaned after each animal. A discrimination index was calculated as *d* = *d1*/*e1* where *d1* is the time spent exploring the novel object minus time spent exploring the familiar object, and *e1* is the total exploration time during training for two identical objects [103].

### 2.7. Excision of the Brain Structures

In both sets of experiments, mice were decapitated 46–48 h after behavioral testing (at 12:00–14:00 p.m.). Brains were excised on ice, and the entire frontal cortex, hippocampus, and midbrain were dissected according to coordinates from the online mouse brain atlas (https://scalablebrainatlas.incf.org/mouse/ABA_v3, accessed on 11 October 2021), then frozen in liquid nitrogen, and stored at −80 °C until total-RNA or total-protein isolation procedures.

### 2.8. Reverse-Transcription Quantitative PCR

Total RNA was extracted using the ExtractRNA reagent (Evrogen, Moscow, Russia) according to the manufacturer’s instructions, treated with RNA-free DNase (Promega, Madison, WI, USA), and diluted to 0.125 μg/μL with diethyl pyrocarbonate–treated water. One microgram of total RNA was subjected to cDNA synthesis with a random hexanucleotide mixture [104,105]. The numbers of cDNA copies for gene encoding DNA-dependent RNA polymerase 2 subunit A (*Polr2a*), BDNF, receptors TrkB and p75, TH, DAT, MAOA, COMT, and DA receptors D1 and D2 were determined with quantitative PCR on LightCycler 480 (Roche Applied Science) using selective primers (Table A1), SYBR Green I fluorescence detection (R-414 Master mix, Syntol, Moscow, Russia), and genomic DNA extracted from the livers of male C57BL/6 mice as the external standard. We utilized 50, 100, 200, 400, 800, 1600, 3200, and 6400 copies of genomic DNA/μL as external standards for all genes under study. Reagent controls were set up under the same conditions but without the template. Gene expression was evaluated as the number of cDNA copies per 100 copies of *Polr2a* cDNA [104,105,106]. Melting-curve analysis was performed at the end of each run for each primer pair, allowing us to control the amplification specificity.

### 2.9. Western Blot

For assessment of total protein levels, tissue was homogenized in LB buffer (300 mM NaCl, 100 mM Tris-HCl pH 8, 4 mM EDTA, 0.2% of Triton X-100, 1 mM NaVO_4_, 2 mM PMSF, and a protease inhibitor cocktail), incubated for 60 min on ice, and centrifuged (12,000× *g*, 15 min). The supernatant containing total protein was transferred to a clean tube and kept at −80 °C. Protein concentration was estimated using the Pierce BCA Protein Assay Kit (Thermo Fisher Scientific Inc., Waltham, MA, USA) on a NanoDrop 2000C spectrophotometer (Thermo Fisher Scientific Inc., Waltham, MA, USA) followed by the adjustment of the samples to an equal concentration with a 2× Laemmli buffer. The protein samples were denatured by boiling for 10 min at 95 °C. The extracts (30 μg of total protein per lane for BDNF and pro-BDNF analysis and 15 μg of total protein per lane for the analysis of other proteins: TH, COMT, D1R, D2R, p75^NTR^, and TrkB) were resolved using SDS-PAGE and blotted onto a nitrocellulose membrane (Bio-Rad Laboratories, Inc., Hercules, CA, USA) by means of a Trans-Blot Turbo Transfer System (Bio-Rad Laboratories, Inc., USA). The membranes were blocked in a Tris-buffered saline supplemented with 0.05% of Triton X-100 (TBST) and containing 5% of nonfat dry milk (NFDM-TBST) for 1 h, rinsed, and then incubated with primary antibodies (Table 1). After protein detection (as described below), all blots were stripped and then reprobed with anti-GAPDH as a loading control. For protein detection, membranes were washed in TBST (5 × 5 min), followed by incubation with a secondary antibody conjugated with horseradish peroxidase. After washing, the blots were treated with Clarity Western ECL Substrate (Bio-Rad Laboratories, Inc., USA) according to the manufacturer’s instructions. The protein bands were detected on a C-DiGit Blot Scanner (LI-COR, Lincoln, NE, USA). The quantification of protein bands was performed using the ImageStudio software (LI-COR Image Studio Software, Lincoln, NE, USA). The target protein levels were normalized to GAPDH levels.

### 2.10. Fluorescence Microscopy of Mouse Brain Sections

Mice were transcardially perfused with a phosphate-buffered saline (PBS) and a 4% paraformaldehyde solution under anesthesia 6 weeks after the rAAV injection. The brains were removed and post-fixed with 4% paraformaldehyde for 6 h and immersed in 30% sucrose in PBS for 2 days. Sequential 14-µm slices were prepared on a cryostat (Thermo Scientific, Waltham, MA, USA). The cell nuclei were stained with a bis-benzimide solution (Hoechst 33258 dye, 5 µg/mL in PBS, Sigma-Aldrich, Darmstadt, Germany). Finally, the sections were mounted in an antiquenching medium (Fluoromount G; SouthernBiotech, Birmingham, AL, USA), followed by microscopy under a Zeiss AxioImager2 microscope with 10× and 40× air-immersion objectives.

### 2.11. Statistics

The data are shown as the mean ± SEM. The Dixon’s Q test was performed to identify outliers and remove them from a dataset. After testing for the Gaussian distribution by the D’Agostino–Pearson normality test, datasets were compared either by two-tailed Student’s *t* test or by the nonparametric Mann–Whitney *U* Test. The statistical significance was set to *p* < 0.05.

## 3. Results

### 3.1. The i.c.v. Injection of BDNF Failed to Affect Behavior in BTBR Mice

The total distance traveled (*t* = 0.23, *p* = 0.81), explored area of the arena (*U* = 0.46, *p* = 0.79), time in the center of the arena (*t* = 0.16, *p* = 0.87), and the number of rearings (*t* = 0.36, *p* = 0.71) did not change in the open field test after i.c.v. injection of the recombinant BDNF protein (Table 2). These findings indicated that both locomotor and exploratory activities were not affected by the i.c.v. injection of BDNF.

It was found that time in the open (*t* = 0.61, *p* = 0.54) and closed (*t* = 0.77, *p* = 0.44) arms and the explored area of open (*U* = 43, *p* = 0.63) and closed (*U* = 44, *p* = 0.96) arms were not altered in the experimental group as compared to the control (Table 3). The total distance traveled (*t* = 0.91, *p* = 0.37) and the number of head-dips (*t* = 0.76, *p* = 0.45) were not affected by the BDNF protein injection either. Thus, there was no effect of i.c.v. BDNF protein administration on anxiety-related behavior.

**Figure 2:**
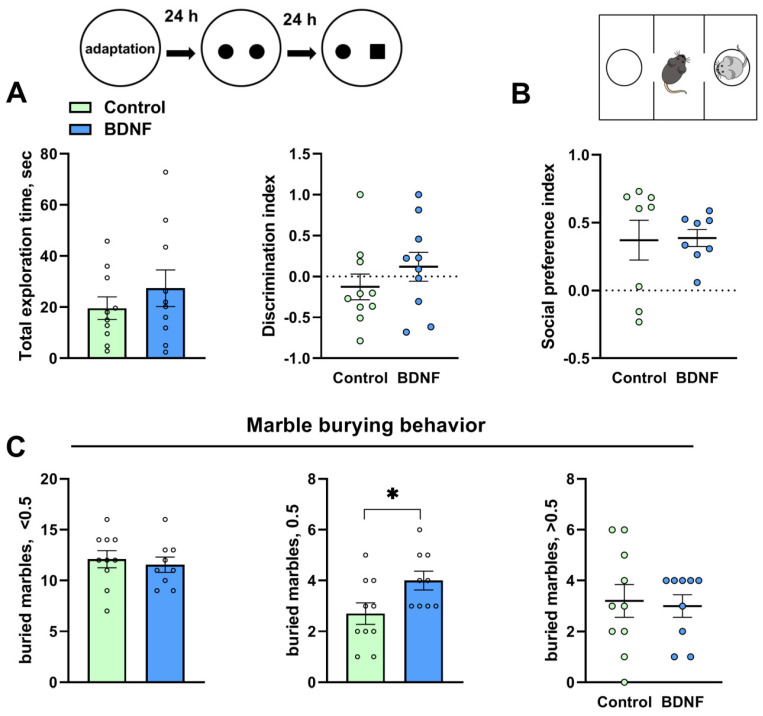
Effects of BDNF i.c.v. administration to BTBR mice on the novel object recognition (**A**), social preference in the three-chambered social approach test (**B**), and stereotypy (**C**). All the values are presented as mean ± SEM; *n* ≥ 8. * *p* ˂ 0.05 vs. a control.

The total exploration time (*t* = 0.92, *p* = 0.36) and the discrimination index (*t* = 1.04, *p* = 0.31) were unaltered in the novel object recognition test after the i.c.v. injection of BDNF (Figure 2A). The social preference index was unchanged (*U* = 24, *p* = 0.44) in the animals treated with BDNF protein (Figure 2B). The number of marbles buried at half depth was significantly higher in the group treated with BDNF (*t* = 2.28, *p* = 0.03; Figure 2C); however, the numbers of marbles buried by less than a half and by more than a half were unchanged (*t* = 0.48, *p* = 0.63 and *U* = 26.50, *p* = 0.36, respectively).

### 3.2. mRNA and Protein Levels of Key Genes of the DA System Are Changed by the i.c.v. Injection of BDNF

**Figure 3:**
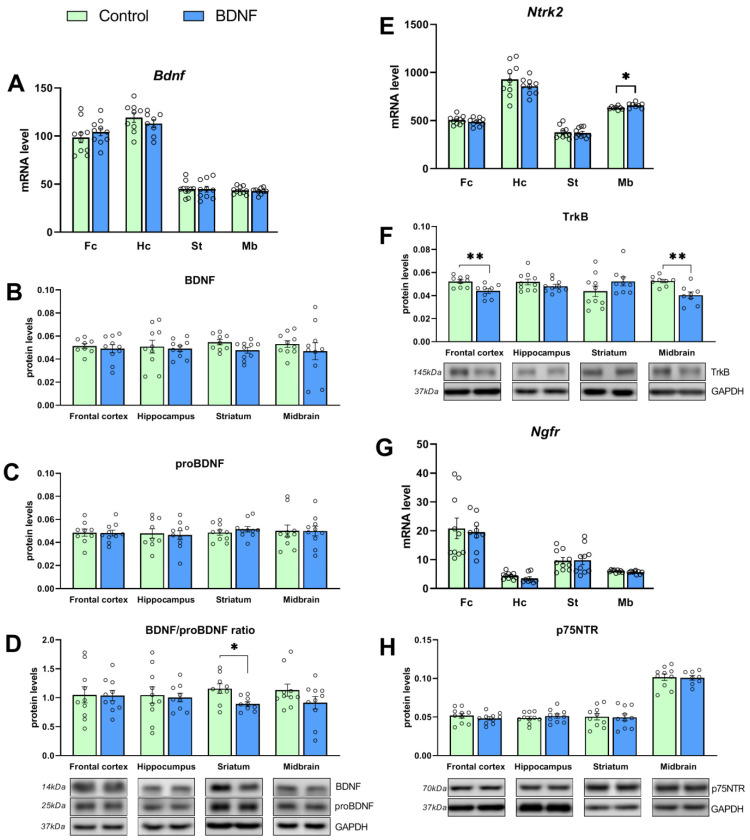
(**A**–**H**) The impact of BDNF i.c.v. administration on mRNA levels of genes *Bdnf*, *Ntrk2*, and *Ngfr* (**A**,**E**,**G**) and protein levels of BDNF, proBDNF, TrkB, and p75NTR in various brain structures of BTBR mice. Fc: frontal cortex, Hc: hippocampus, Mb: midbrain, St: striatum. Gene expression is presented as the number of a gene’s cDNA copies per 100 cDNA copies of *rPol2*. Protein levels are indicated as the ratio of chemiluminescence intensity of a target protein to that of GAPDH. All the values are presented as mean ± SEM; *n* ≥ 8. * *p* ˂ 0.05, ** *p* ˂ 0.01 vs. a control.

We did not find any changes in the *Bdnf* mRNA level in any of the studied brain structures (*t* = 0.85, *p* = 0.40; *t* = 0.95, *p* = 0.35; *t* = 0.07, *p* = 0.94; and *t* = 0.44, *p* = 0.66 for the hippocampus, frontal cortex, striatum, and midbrain, respectively; Figure 3A). At the same time, an increase in *Ntrk2* (encoding TrkB receptor) transcription was detected in the midbrain (*t* = 2.26, *p* = 0.04; Figure 3E) but not in the frontal cortex (*t* = 0.00, *p* = 0.99), hippocampus (*t* = 1.09, *p* = 0.28), and striatum (*t* = 0.21, *p* = 0.83). The *Ngfr* (encoding receptor p75) mRNA level (*t* = 0.30, *p* = 0.76; *U* = 0.19, *p* = 0.06; *t* = 0.08, *p* = 0.93; and *t* = 1.67, *p* = 0.11 for the hippocampus, frontal cortex, striatum, and midbrain, respectively; Figure 3G) was also unaltered in all the tested brain structures.

No changes were found in the protein levels of both proBDNF and mature BDNF in all the investigated brain structures (Figure 3B,C). By contrast, the proBDNF/BDNF ratio proved to be reduced (*t* = 2.89, *p* = 0.01) in the striatum of mice treated with the BDNF protein (Figure 3D). We noted a decrease in the TrkB receptor protein level in the frontal cortex (*t* = 3.2, *p* = 0.005) and midbrain (*t* = 3.72, *p* = 0.002) of mice from the experimental group (Figure 3F). At the same time, the central BDNF administration failed to alter the p75^NTR^ protein level (Figure 3H) in all the studied brain structures.

**Figure 4:**
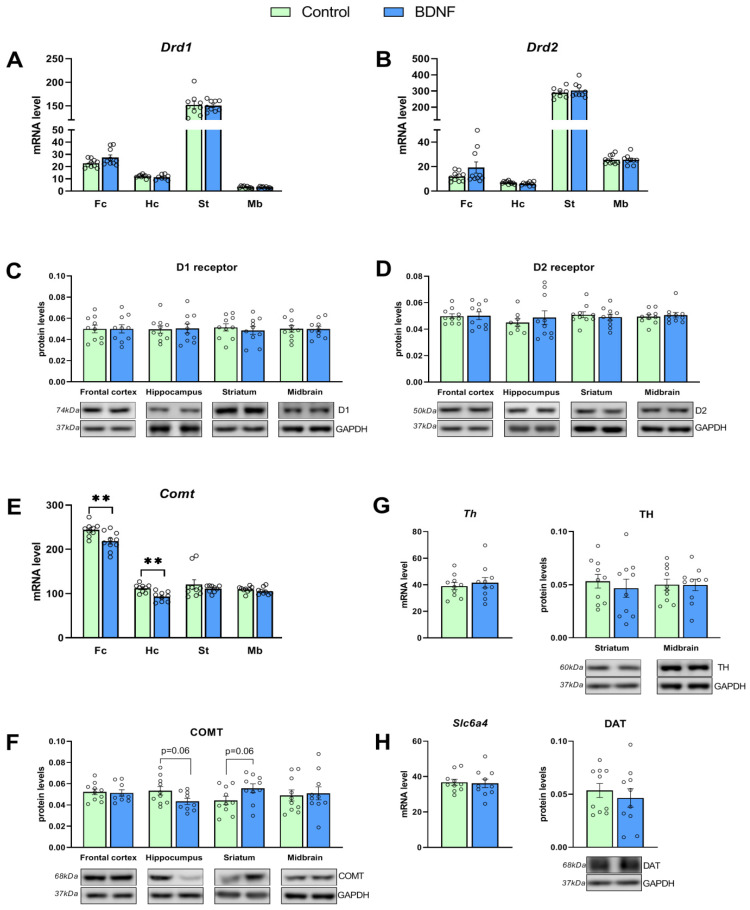
Effects of the BDNF i.c.v. administration on mRNA levels of genes *Drd1*, *Drd2*, *Comt*, *Th*, and *Slc6a4* (**A**,**B**,**E**,**G**,**H**) and protein levels of DA receptors D1 and D2, COMT, TH, and DAT (**C**,**D**,**F**–**H**) in various brain regions of BTBR mice. Gene expression is presented as the number of a gene’s cDNA copies per 100 cDNA copies of *rPol2*. Protein levels are illustrated ratios of chemiluminescence intensity of a target protein to that of GAPDH. All the values are mean ± SEM; *n* ≥ 8. ** *p* ˂ 0.01 vs. a control.

Assays of mRNA expression of DA system genes did not reveal any changes in *Drd1* transcription (*U* = 29, *p* = 0.12; *t* = 0.96, *p* = 0.35; *U* = 0.38, *p* = 0.86; and *t* = 0.69, *p* = 0.49 for the hippocampus, frontal cortex, striatum, and midbrain, respectively; Figure 4A) and *Drd2* transcription (*t* = 1.54, *p* = 0.13; *t* = 1.54, *p* = 0.14; *t* = 0.73, *p* = 0.47; and *t* = 0.003, *p* = 0.99 for the hippocampus, frontal cortex, striatum, and midbrain, respectively; Figure 4B). Meanwhile, the *Comt* mRNA level turned out to be reduced in the frontal cortex (*t* = 3.03, *p* = 0.007) and hippocampus (*t* = 4.01, *p* = 0.001) of the experimental mice (Figure 4E). No changes in *Comt* transcription in the striatum (*t* = 0.84, *p* = 0.40) and midbrain (t = 1.16, *p* = 0.26) were detectable. Furthermore, the central BDNF administration failed to affect the midbrain mRNA levels of genes *Th* (*t* = 0.53, *p* = 0.59) and the *Slc6a4* (*t* = 0.20, *p* = 0.84) encoding of the TH enzyme and DAT, respectively (Figure 4G,H).

We did not notice any influence of the BDNF injection on protein levels of DA receptors D1 and D2 in all investigated brain structures (Figure 4C,D), of TH (Figure 4G) in the striatum and midbrain, and of DAT in the striatum (Figure 4H). In the meantime, a trend toward a decrease in the COMT enzyme protein level in the hippocampus (*t* = 2.00, *p* = 0.06) and striatum (*t* = 2.01, *p* = 0.059; Figure 4F) was revealed.

### 3.3. rAAV-Mediated Bdnf Gene Delivery Effectively Overexpresses BDNF in the Hippocampus of BTBR Mice

**Figure 5:**
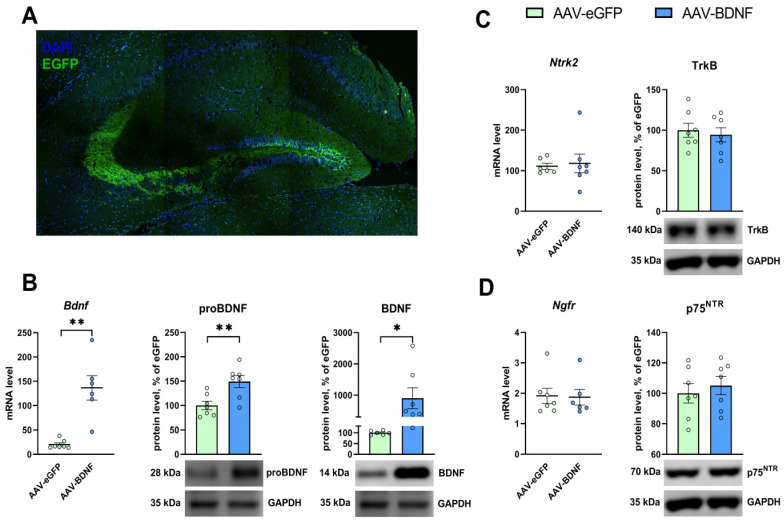
(**A**) A representative micrograph of a brain slice after AAV-EGFP injection. Hippocampal areas virally transduced with the rAAV vector are visible. (**B**) The mRNA expression of *Bdnf* and protein levels of proBDNF and BDNF in the hippocampus of BTBR mice. The mRNA and protein levels of TrkB (**C**) and p75^NTR^ (**D**) in the hippocampi of BTBR mice featuring BDNF overexpression and of the control animals. Each mRNA level is presented as the number of a gene’s cDNA copies divided by 100 cDNA copies of *rPol2*. Protein levels are illustrated as the ratio of chemiluminescence intensity of a target protein to that of GAPDH. Data are given as mean ± SEM; *n* ≥ 6. * *p* < 0.05, ** *p* < 0.01 vs. the AAV-eGFP group.

The intrahippocampal administration of rAAVs carrying pAAV-Syn-Bdnf-eGFP resulted in significant elevation of BDNF expression. The mRNA level of *Bdnf* dramatically went up (*U* = 0, *p* = 0.0012) in the hippocampi of mice in the experimental group (Figure 5B). The protein levels of BDNF and of its precursor proBDNF significantly increased, too, (*t* = 2.19, *p* = 0.05, and *t* = 3.26, *p* = 0.006) in the animals injected with AAV-BDNF (Figure 5B).

### 3.4. Hippocampal BDNF Overexpression Reduces Anxiety and Stereotypy in BTBR Mice

**Figure 6:**
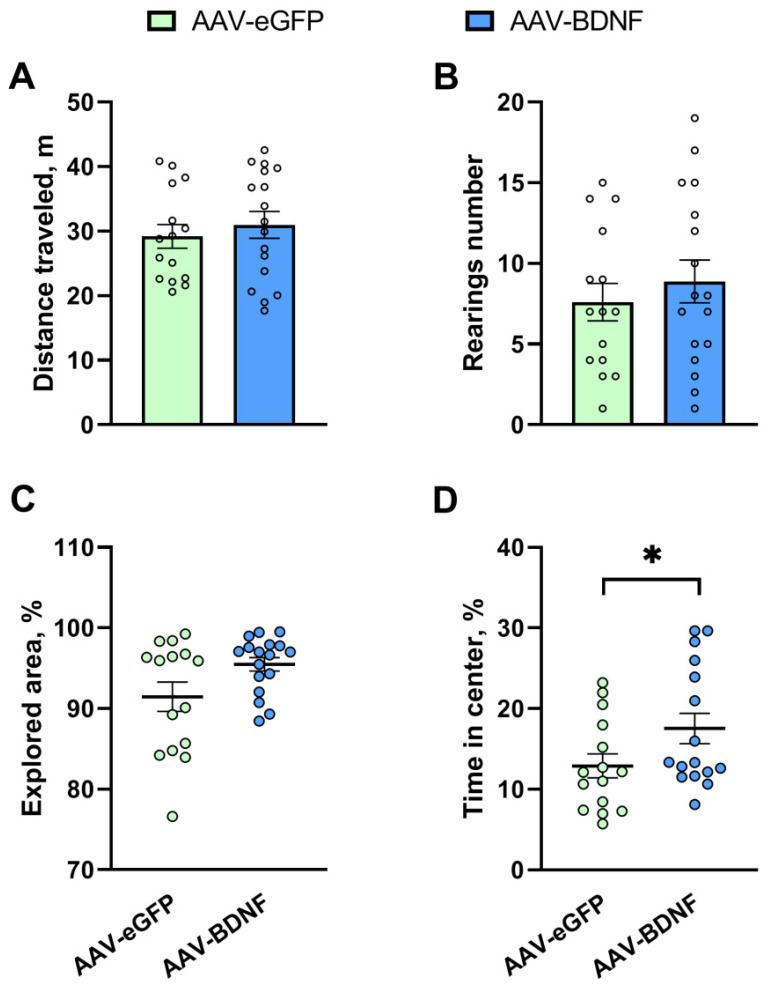
The influence of BDNF overexpression in the hippocampus of BTBR mice on total distance traveled (**A**), on the rearing number (**B**), on explored area of the arena (**C**), and on time spent in the center of the arena (**D**) in the open field test. All the values are presented as mean ± SEM. *n* ≥ 15. * *p* ˂ 0.05 vs. the AAV-eGFP group.

We observed a significant increase (*U* = 70, *p* = 0.04) in the time spent in the center of the arena in the open field test in mice with hippocampal BDNF overexpression (Figure 6D). At the same time, the total distance traveled (*t* = 0.64, *p* = 0.53), the explored area of the arena (*U* = 82, *p* = 0.09), and the number of rearings (*t* = 0.72, *p* = 0.47) were unchanged in mice from the experimental group (Figure 6A–C).

**Figure 7:**
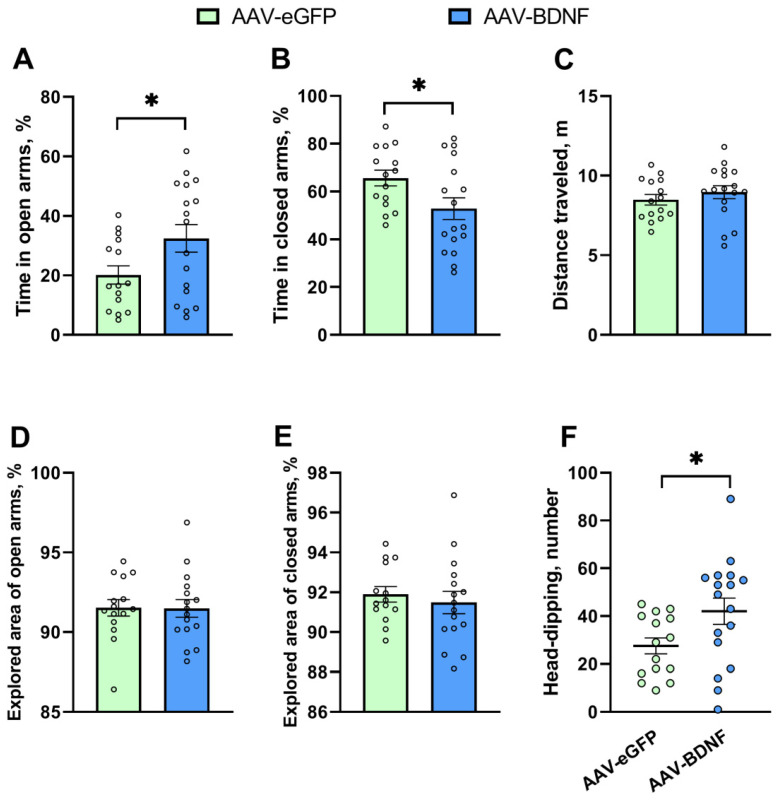
(**A**–**F**) Anxiety-related behavior of BTBR mice in the elevated plus maze test after hippocampal BDNF overexpression. All the values are given as mean ± SEM; *n* ≥ 15. * *p* ˂ 0.05, as compared with the AAV-eGFP group.

In the elevated plus maze, BDNF overexpression extended the time spent in open arms (*t* = 2.14, *p* = 0.04) and simultaneously diminished the time spent in closed arms (*t* = 2.22, *p* = 0.03) (Figure 7A,B). In addition, an increase in the number of head-dips (*U* = 72.5, *p* = 0.03) was detected in the experimental group (Figure 7F). On the contrary, the BDNF overexpression failed to affect the explored area of open (*t* = 0.05, *p* = 0.96) and closed (*t* = 0.57, *p* = 0.56) arms and the total distance traveled (*t* = 0.88, *p* = 0.38) (Figure 7C–E). Taken together, our findings indicated that hippocampal BDNF overexpression exerted an anxiolytic effect in BTBR mice. Of note, the exploratory activity associated with risky behavior (head-dipping) also increased in the mice featuring BDNF overexpression.

**Figure 8:**
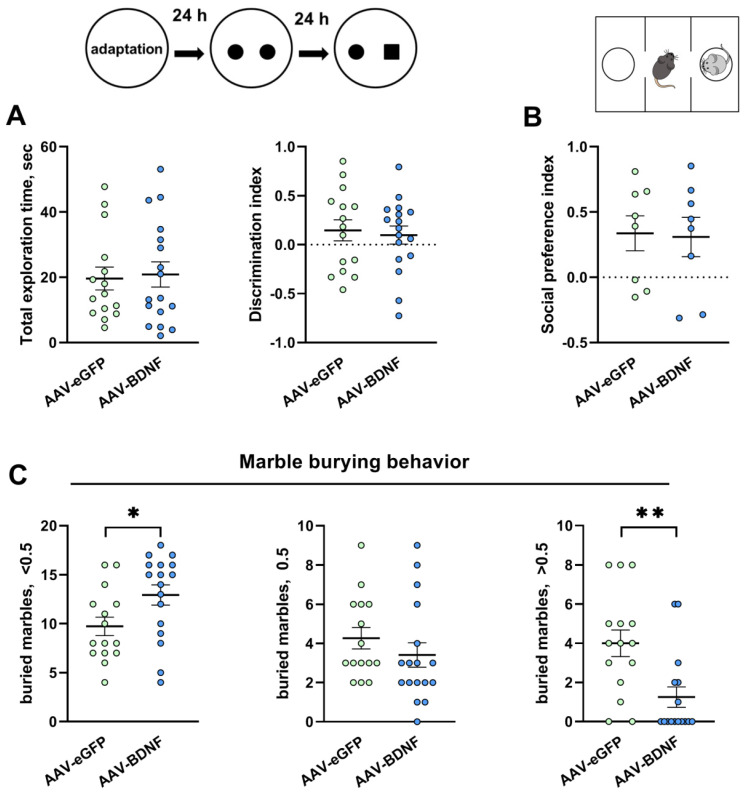
Effects of BDNF overexpression in the hippocampus of BTBR mice on novel object recognition (**A**), on a social preference in the three-chambered social approach test (**B**), and on stereotypy (**C**). All the values are presented as mean ± SEM; *n* ≥ 15. * *p* ˂ 0.05; ** *p* ˂ 0.01 vs. the AAV-eGFP group.

In the animals with BDNF overexpression, neither the total exploration time (*U* = 125.5, *p* = 0.94) nor the discrimination index (*t* = 0.34, *p* = 0.73) was found to be altered in the novel object recognition test (Figure 8A). The social preference index also was unchanged (*t* = 0.13, *p* = 0.89) in the experimental mice (Figure 8B).

The most interesting result is that the number of marbles buried at less than half depth was significantly higher, while the number of marbles buried by more than a half was significantly lower (*U* = 69, *p* = 0.02 and *U* = 48.50, *p* = 0.0028, respectively) in the group featuring BDNF overexpression (Figure 8C). These data suggested that BDNF overexpression in the hippocampus of BTBR mice led to a significant decrease in stereotypical behavior, which is known to be associated with ASDs.

### 3.5. mRNA and Protein Levels in the Hippocampus after the AAV-BDNF Injection

**Figure 9:**
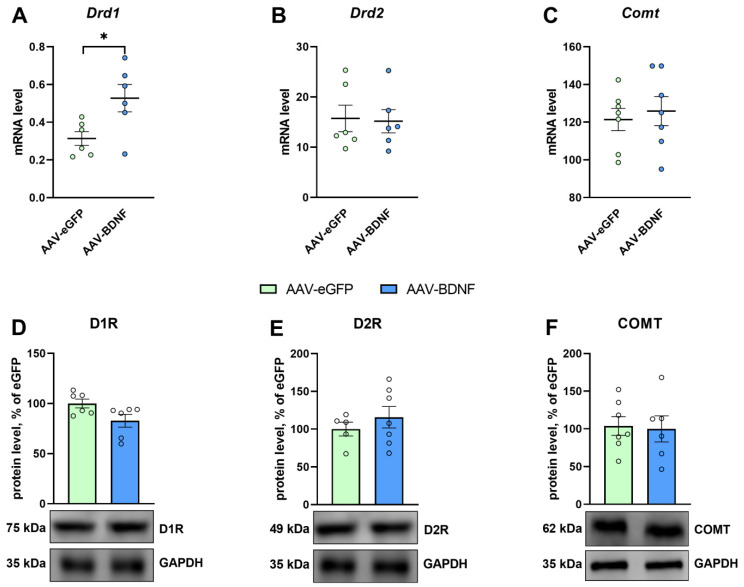
The impact of BDNF overexpression in the hippocampus of BTBR mice on mRNA levels of genes *Drd1, Drd2,* and *Comt* (**A**–**C**) and on protein levels of DA receptors D1 and D2 and COMT (**D**–**F**). Gene expression is presented as the number of a gene’s cDNA copies per 100 cDNA copies of *rPol2*. Protein levels are given as the ratio of chemiluminescence intensity of a target protein to that of GAPDH. All the values are mean ± SEM; *n* ≥ 6. * *p* ˂ 0.05 as compared with the AAV-eGFP group.

The induction of BDNF overexpression in the hippocampus of BTBR mice significantly raised *Drd1* mRNA expression (*t* = 2.62, *p* = 0.02; Figure 9A). Meanwhile, no changes in mRNA levels of genes *Ntrk2* (*t* = 0.27, *p* = 0.79), *Ngfr* (*U* = 19.0, *p* = 0.83) (Figure 5C,D), *Drd2* (*U* = 18.0, *p* = 0.99), and *Comt* (*t* = 0.46, *p* = 0.65) were detectable (Figure 9B,D). Additionally, the hippocampal BDNF overexpression failed to produce any significant changes in the protein levels of TrkB (*t* = 0.45, *p* = 0.65), p75^NTR^ (*t* = 0.58, *p* = 0.56) (Figure 5H,K), receptor D1 (*U* = 10.0, *p* = 0.24), receptor D2 (*t* = 0.84, *p* = 0.42) (Figure 9D,E), and COMT (*t* = 0.18, *p* = 0.86; Figure 9F).

## 4. Discussion

Earlier, we have researched the effect of central BDNF protein administration on different kinds of behavior and on the brain serotonin system. An antidepressant effect was demonstrated at 17 days after acute, central BDNF administration at the same dose as in the present study (300 ng i.c.v.) in ASC mice, which are genetically predisposed to depressive-like behavior. These behavioral changes were accompanied by a significant alteration of the brain serotonin system [107]. Centrally administered BDNF (same dose, 300 ng i.c.v.) also inhibits the marble-burying activity and restores a sexual female preference of adult male offspring that is inverted by prenatal exposure to combined ethanol and stress during gestation; an ameliorative effect is seen in 7–10 days after BDNF administration [108]. A considerably longer-lasting ameliorative action of BDNF has been demonstrated for prepulse inhibition (PPI) disrupted in DBA/2J mice [109]. In that paper, it was revealed that BDNF (same dose, 300 ng, i.c.v.) significantly expanded the amplitude of a startle response and restored disrupted PPI at 7 days after acute administration. A significant BDNF-induced increase in PPI persisted even 1.5 months after single, acute BDNF administration [109].

In the light of these observations, it was unusual to observe here the complete absence of an influence of central BDNF administration on behavior in BTBR mice. Likely, the general increase in BDNF-induced neurogenesis and neurites outgrowth leads to an increase in the already large losses of the signal that results from the dysregulation of postnatal synaptic pruning observed in ASD [87] that may even cause negative consequences with regard to autistic-like behavior. On the other hand, it is well known that transcription factor CREB mediates BDNF’s effects on neurons [110]. Recently, we revealed that a transcription factor *Cc2d1a*/Freud-1 knockdown in the hippocampus of BTBR mice failed to affect CREB transcription factor expression and phosphorylation [111], whereas, in C57BL/6J mice, the *Cc2d1a*/Freud-1 knockdown caused a considerable reduction in both CREB expression and phosphorylation [112]. On the basis of these data, we proposed an impairment of a CREB-dependent pathway in BTBR mice; this mechanism may explain the absence of effects of central BDNF administration on behavior (seen in the present paper).

Nevertheless, previous experiments with hippocampal BDNF overexpression suggest that BDNF does play an important role in the mechanisms underlying anxiety and repetitive stereotypical behavior; these mechanisms are known to be disturbed in ASDs [113,114]. Moreover, anxiety is a reason for active stress avoidance in ASDs, thus considerably affecting quality of life of both autistic patients and their caregivers. Here, BDNF overexpression in the murine hippocampus resulted in a significant increase of the number of marbles buried at less than a half depth and in a decrease in the number of marbles buried by more than a half, indicating amelioration of stereotypical behavior. It is noteworthy that our central BDNF protein administration increased the number of marbles buried by half without affecting other parameters in the marble burying test; these data are hard to interpret. Furthermore, the BDNF overexpression alleviated anxiety as assessed using different tests. Mice of the experimental group demonstrated longer time spent in the center of the arena in the open field test and extended time spent in open arms of the elevated plus maze together with shorter time spent in closed arms of the maze. Recently, it was shown that i.c.v.-injected BDNF can dose-dependently decrease anxiety-like behavior in mice [115]. Likewise, hippocampal BDNF overexpression ameliorates the anxiety of animals [116,117]. It is worth mentioning that in a study by Bahi [118], it was demonstrated that in rats with an autistic-like phenotype, hippocampal BDNF overexpression attenuates anxiety both in the elevated plus maze test and open field test as well as decreases stereotypy in the marble burying test. In addition, mice overexpressing a BDNF transgene in the forebrain neurons are characterized by less severe stereotypical behavior in the marble burying test and reduced anxiety-like behavior in the elevated plus maze test [119]. Thus, our findings are in good concordance with evidence of anxiolytic-like properties of BDNF, including results obtained in animals with an autistic-like phenotype. It could be suggested that the local increase in BDNF expression in the hippocampus partially corrects neuronal deficit in the corpus callosum observed both in ASD patients [95] and BTBR mice [94] that results in the amelioration of anxiety and stereotypical behavior.

There are a lot of data on the crosstalk between BDNF and DA systems in the brain. BDNF enhances the survival of dopaminergic neurons in mesencephalic cultures and a developing SN. BDNF seems to serve as a trophic factor for mesencephalic dopaminergic neurons by increasing their survival, including survival of the neuronal cells that degenerate in Parkinson’s disease [48]. BDNF has even been suggested as a promising therapeutic agent for Parkinson’s disease [120]. Moreover, BDNF enhances a DA release [121] and functional activity of the DA brain system [122]. The essential role of BDNF in the mesolimbic DA pathway has been documented in the social defeat stress model [123]. In BTBR mice, which are characterized by autistic-like behavioral traits, central BDNF administration here diminished the *Comt* mRNA level in the frontal cortex and hippocampus, although a reduction in hippocampal and an increase in striatal COMT protein levels were below the significance threshold. Taking into account the ability of BDNF to increase the DA level and metabolism in the striatum [122], even a minor elevation of striatal COMT protein amounts may indicate a slight enhancement of the activity of the DA system. In turn, hippocampal BDNF overexpression elevated the DA receptor D1 mRNA level that was not accompanied by protein level changes. Taken together, these data implicate the crosstalk between BDNF and DA systems in the mechanisms behind autistic-like behavior. This supposition is partially confirmed by the BDNF-induced decrease in the BDNF/proBDNF ratio in the striatum, one of the key brain structures of the DA system. On the other hand, this reduction in the BDNF/proBDNF ratio probably reflects a compensatory change in endogenous BDNF metabolism after the injection of an excessive amount of the exogenous protein. This finding is in agreement with the downregulation of receptor TrkB in the frontal cortex and midbrain. Such an overall decrease in the functioning of the BDNF system may explain the absence of effects of the BDNF protein on the behavior of BTBR mice, which is in line with evidence of TrkB underexpression in the dorsal striatum of D2^+/−^ knockout mice exposed to early postnatal stress [66].

## 5. Conclusions

Our findings point to the involvement of BDNF and of crosstalk between BDNF and the DA system in the mechanisms underlying autistic-like behavior, because, in the present work, the hippocampal BDNF overexpression exerted an anxiolytic action and reduced stereotypical behavior in BTBR mice. Moreover, taking into account behavioral and neuroanatomical similarities of this animal model and ASD in humans, we believe that BDNF can be considered a target for autism treatment. Nevertheless, a comparison of our BDNF protein i.c.v. injection and BDNF overexpression clearly indicates that only excessive sustained BDNF production can ameliorate autistic-like behavior. For this reason, interventions resulting in a transient BDNF level increase, e.g., physical exercise, diet, social activity, and education (which can reduce long-term risk of cognitive impairment and dementia [124,125,126]), probably will be insufficient for ASD patients. It could be suggested that local intracerebral infusions of BDNF or continuous systemic injections of ligands for BDNF receptors may be favorable. In conclusion, a growing number of studies suggest that different brain neurotransmitter systems play an important part in autistic-like behavior, thereby giving hope that some currently prescribed drugs affecting neurotransmission may become ASD treatments.

## Figures and Tables

**Table 1:** Characteristics of antibodies used.

Target Protein	Primary Antibody		Secondary Antibody: Dilution, Manufacturer Code
	Antibody Dilution	Manufacturer Code	
BDNF	1:1000	Ab108319 (Abcam, UK)	Anti-rabbit 1:10,000, G-21234 (Invitrogen, USA)
TrkB	1:400	Ab32096 (Abcam, UK)	
P75^NTR^	1:500	Ab38335 (Abcam, UK)	
TH	1:500	Ab112 (Abcam, UK)	
DAT	1:500	Ab184451 (Abcam, UK)	
COMT	1:200	Sc-137253 (Santa Cruz, USA)	
D1R	1:250	Ab20066 (Abcam, UK)	
D2R	1:500	Sc-9113 (Santa Cruz, USA)	
proBDNF	1:2500	Sc-65513 (Santa Cruz, USA)	Anti-mouse 1:30,000, #31430 (Invitrogen, Waltham, MA, USA)
GAPDH	1:7000	Ab8245 (Abcam, UK)	Anti-mouse 1:30,000, ab6728 (Abcam, Cambridge, UK)

**Table 2:** Behavior of BTBR mice in the open field test after i.c.v. injection with BDNF.

Parameter	Control	BDNF	*p* Values
Time in the center of arena, %	15.88 ± 1.83	15.49 ± 1.54	*p* = 0.87
Explored area of the arena, %	94.35 ± 2.00	95.82 ± 1.21	*p* = 0.79
Distance traveled, m	34.70 ± 3.61	35.74 ± 2.54	*p* = 0.81
Rearings, number	16.30 ± 2.64	17.80 ± 3.08	*p* = 0.71

Data “Explored area of the arena” were compared using the nonparametric Mann–Whitney U Test, other parameters were compared using two-tailed Student’s *t* test.

**Table 3:** Behavior of BTBR mice in the elevated plus maze test after i.c.v. injection with BDNF.

Parameter	Control	BDNF	*p* Values
Time in open arms, %	19.26 ± 4.46	23.11 ± 4.41	*p* = 0.55
Explored area of open arms, %	62.04 ± 9.83	67.93 ± 8.88	*p* = 0.63
Time in closed arms, %	67.16 ± 5.16	61.61 ± 4.91	*p* = 0.45
Explored area of closed arms, %	91.24 ± 0.49	91.16 ± 0.39	*p* = 0.97
Distance traveled, m	8.36 ± 0.52	8.98 ± 0.43	*p* = 0.37
Head-dips, number	20.40 ± 4.08	24.70 ± 3.86	*p* = 0.45

Explored area of open and closed arms were compared using the nonparametric Mann–Whitney U Test, other parameters were compared using two-tailed Student’s *t* test.

## Data Availability

Data are available from the corresponding author upon reasonable request.

## Appendix A

**Table A1:** The primer sequences, annealing temperatures, and PCR products’ lengths.

Target Gene	Primer Sequences	Annealing Temperature, °C	Amplicon Length, bp
*rPol2*	F 5′-TGTGACAACTCCATACAATGC-3′ R 5′-CTCTCTTAGTGAATTTGCGTACT-3′	60	188
*Bdnf*	F 5′-TGAAGCCACCTCTCTCAGTC-3′ R 5′-AGCAGTACTCTCAGGGCTAGG-3′	59	126
*Ngfr*	F 5′-CTTGCTTCTGACCACCCTTCTC-3′ R 5′-GCTCCCTTCTCCTAGTGTCAAAC-3′	61	145
*Ntrk2*	F 5′-GTCGCTTTCTCTCCCTTTGG-3′ R 5′-GCATTGTTAGTTGTGGTGGGC-3′	60	277
*Drd1*	F 5′-GGAAACCCTGTCGAATGCTCTC-3′ R 5′-CCAGCCAAACCACACAAATACATCG-3′	64	222
*Drd2*	F 5′-TCCGCCACTTCTTGACATACATTG-3′ R 5′-CCCATCCACAGCCTCCTCTAAG-3′	65	203
*Th*	F 5′-CCGTACACCCTGGCCATTGATG-3′ R 5′-ATGAAGGCCAGGAGGAATGCAGG-3′	64	320
*Slc6a3*	F 5′-TTCACTGTCATCCTCATCTCTTTC-3′ R 5′-TCAAAATACTCAGCAGCGGGTG-3′	63	224
* Comt *	F 5′-GACTTCCTGGCGTATGTGAG-3′ R 5′-AGAGTGAGTGTGTGTCATCG-3′	60	199
* Maoa *	F 5′-AATGAGGATGTTAAATGGGTAGATGTTGGT-3′ R 5′-CTTGACATATTCAACTAGACGCTC-3′	61	138
